# Discriminant Analysis of *Undaria pinnatifida* Production Areas Using Trace Elemental Analysis

**DOI:** 10.1155/2010/149895

**Published:** 2010-05-12

**Authors:** Mikio Kaihara

**Affiliations:** Department of Chemical Engineering, Ichinoseki National College of Technology, Hagisho, Ichinoseki, Iwate 021-8511, Japan

## Abstract

Increasingly, attention is being paid to declaring the origin of agricultural and marine products after the advent of the bovine spongiform encephalopathy (BSE; commonly known as mad-cow disease). The display of the production centers on *U. pinnatifida* has been required in Japan since 2006. As an example of testing in another marine product, near-infrared spectra (NIR) and trace elemental analysis of *U. pinnatifida* are proven effective methods for discriminating production centers by us and Food and Agricultural Materials Inspection Center (FAMIC). In the present study, we found that X-ray fluorescence analysis of Br was also effective for the discrimination of production centers. The results of our study suggest that a combination of NIR and X-ray fluorescence analysis is a convenient and efficient method for determination due simple sampling procedures and increased effectiveness.

## 1. Introduction

Issues related to the safety of foods have gathered the attention of consumers following the advent of bovine spongiform encephalopathy (BSE). There have also been improper displays of production centers on some marine products such as clams (*Undaria pinnatifida*). Hence, there is an urgent need for establishment of convenient and efficient scientific methods for discriminating the production centers.

In our earlier studies [1, 2], we reported that near-infrared spectroscopy is a useful method for discriminating production areas [3]. In addition, Food and Agricultural Materials Inspection Center (FAMIC) reported that the inorganic elemental analysis results were valid markers for discriminating the production areas between China, Korea, and Japan (Sanriku and Naruto) of *U. pinnatifida *by ICP-MS spectroscopy [4–7]. Analysis results of these studies showed that the error rates were 0%, 26%, and 6% for samples from China, Korea, and Japan, respectively. In our present study, we hypothesize that additional accuracy can be achieved by classification and regression trees (CART [8, 9]) as a discriminant method.

In addition we explored the possibility of a more convenient discrimination method using elemental analysis of Br. The main purpose of this paper is reporting the possibility of the screening test, which would be conveniently applicable without using expensive and large equipments like an ICP-MS and so on, for discriminating production areas. Some NIR or X-ray fluorescence equipments are portable and the usages are not so complicated.

## 2. Materials and Methods

Results from the study in [5] involving ICP-MS analysis for 22 elements, namely, Al, Ba, Ca, Fe, K, Mg, Mn, Sr, Li, Co, Ni, Cu, Zn, Rb, Y, Mo, Cd, La, Nd, Sm, Gd, and W in 95 *U. pinnatifida* samples (29 China, 19 Korea, and 47 Japan (21 Sanriku and 26 Naruto)) were taken in our study for comparison of linear discriminant analysis (Table 1) and CART (Table 2). The overview of the sample preparation is below as follows:

1. cleaning samples with the ion-exchanged pure water thoroughly for getting rid of attached salt and the others,
2. drying them in the shade for one day under 20 degrees centigrade,
3. drying them in a vacuum with the pressure, about 5 mm Hg, under 107 degrees centigrade for one hour,
4. milling the dried samples into a fine small powder of less than 125 micrometers using a food processor (Millser IFM-700 G, Iwatani Corp.).

Sample preparation and analysis conditions for ICP-MS of the study are described in [5]. 

A total of 10 samples were independently taken in the present study: 3 samples each from China and Korea and 4 samples from Japan (2 each from Sanriku and Naruto). Samples for X-ray fluorescence analysis were prepared according to the method described in [10]. A Shimadzu XRF-1800 was used for detecting trace elements by fundamental parameter methods. Sample briquettes were formed under 20 ton/cm2 pressure for 30 seconds with the MP-35-02 press. 

Because we supposed that there could be the possibility of finding more convenient methods with using an equipment such as an X-ray fluorescence analysis, we gathered independent 10 samples from Riken Food Company.

## 3. Results and Discussion

### 3.1. CART Results and Comparison to Linear Discriminant Analysis

At first, we introduce the overview of how CART works. 

In case of CART, if the target variable is categorical, then a classification tree is generated. To predict the category of the target variable using a classification tree, use the values of the predictor variables to move through the tree until you reach a terminal (leaf) node, then predict the category shown for that node. An example of a classification tree is known well in case of the Fisher's Iris data (from the UCI Machine Learning Repository: Iris Data Set [11]). The target variable is “Species”, the species of Iris. We can see from the tree that if the value of the predictor variable “petal length” is less than or equal to 2.45 the species is Setosa. If the petal length is greater than 2.45, then additional splits are required to classify the species. Because CART is a nonparametric classification method, it is necessary to validate the obtained model. However, CART is usually more potent than LDA.

On the other hand, Linear discriminant analysis is a linear and parametric method with discriminating character. LDA focuses on finding optimal boundaries between classes. 

**Figure 1:**
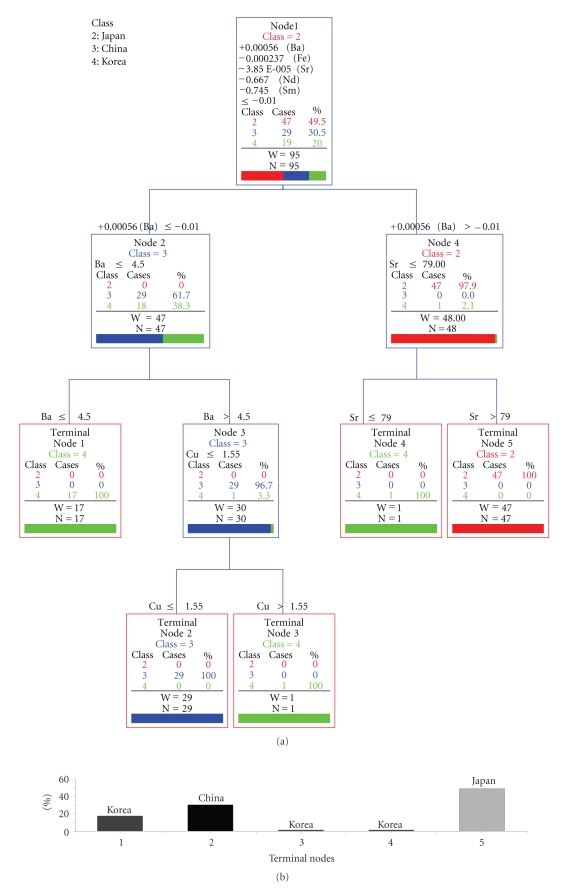
Classification of production areas by elemental analysis.

Classification results between China, Korea, and Japan by CART are shown in Figure 1. Parent and terminal nodes had 2 and 1 minimum cases, respectively.

–In node 1, the discriminant condition is given by the following equation.

(1) $$\begin{array}{cc} & 5.6\times 10_{-4}\times \text{Ba}-2.370\times 10_{-4}\\ & \;\times \text{Fe}-3.850\times 10_{-5}\times \text{Sr}-0.6670\\ & \;\times \text{Nd}-0.7450\times \text{Sm}\;\leq -0.0100. \end{array}$$

–In node 2, the discriminant condition is given by Ba  ≤4.500.
–In node 3, the discriminant condition is given by Cu  ≤1.550.
–In node 4, the discriminant condition is given by Sr  ≤79.00.

**Figure 2:**
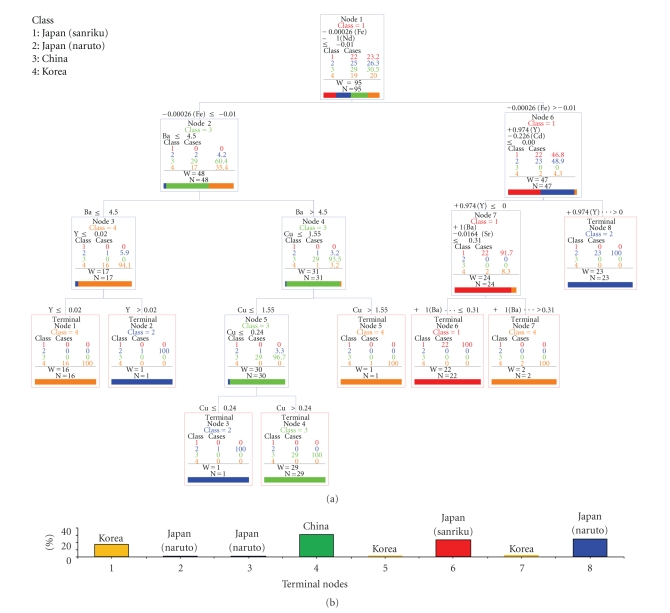
Classification of 4 production areas by elemental analysis.

In the event of the inclusion of 1 case of terminal node 3 and terminal node 4 in China and Japan, respectively, the maximum rate of error was 0/95~2/95. The ranking of variable importance was arranged in descending order, Nd, La, Fe, Sm, Al, Y, Gd, Cd, Ba, Cu, Li, and Sr. Rare earth elements, Fe, and Al were ranked as important. The classification results between Sanriku (Japan), Naruto (Japan), China, and Korea are shown in Figure 2.

–In node 1, the discriminant condition is given by the following equation:

(2) $$-2.000\times 10_{-4}\times \text{Fe}-1.0000\times \text{Nd}\leq -0.0100.$$

–In node 2, the discriminant condition is given by Ba ≤4.500.
–In node 3, the discriminant condition is given by Y  ≤0.0200.
–In node 4, the discriminant condition is given by Cu  ≤1.550.
–In node 5, the discriminant condition is given by Cu  ≤0.240.
–In node 6, the discriminant condition is given by 0.974 × Y − 0.226 × Cd  ≤0.000.
–In node 7, the discriminant condition is given by Ba − 0.0164 × Sr  ≤0.310.

In the event of inclusion of 1 case each of terminal nodes 2, 3, 5, and 7 for samples from Korea, China, China, and Sanriku, respectively, the maximum rate of error was 0/95~5/95. The ranking of variable importance was arranged in descending order: La, Nd, Fe, Y, Sm, Al, Cd, Gd, and Ba. Rare earth elements, Fe, and Al were ranked as important. 

Based on the above CART method, more on the production centers could be extracted by the ICP-MS analysis results of Kadowaki and Tatsuguchi [4] and the proceeding for the discrimination of production centers of *U. pinnatifida*, FAMIC [5], than from the previous linear discriminant analysis. 

### 3.2. Analysis Results by the CART on the Elemental Analysis with an X-Ray Fluorescence Method

**Figure 3:**
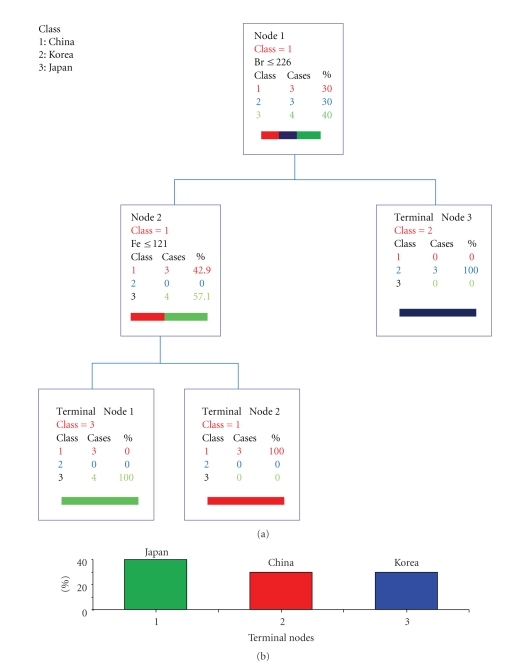
Classification by elemental analysis of Br and Fe.

The classification results from *U. pinnatifida *collected in China, Korea, and Japan based on CART with analysis of 9 major detectable elements detected by X-ray fluorescence method [10] (Figure 3)—Fe, I, Br, As, Zn, Mn, Cu, Ni, and Cr—are given as follows.

–In node 1, the discriminant condition is given by the following equation: Br ≤226.0.
–In node 2, the discriminant condition is given by the following equation: Fe  ≤121.0.

**Figure 4:**
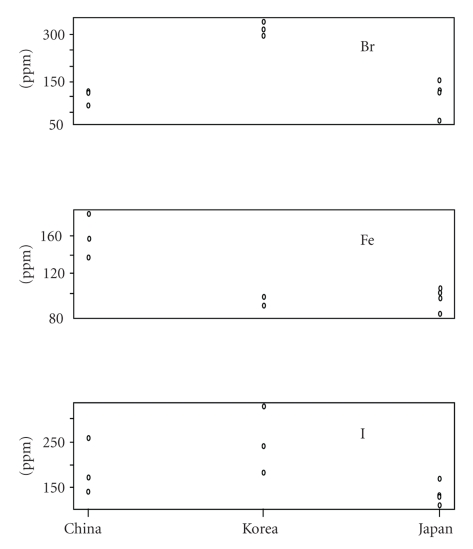
Amount of Br, Fe, and I by country with the X-ray fluorescence.

Terminal nodes 1, 2, and 3 mapped Japan, Korea, and China, respectively. These results suggest that bromine is an important parameter for the discrimination of production centers (characteristic of Korea samples, see Figure 4). Significant differences between Br content for China and Korea samples and between Japan and Korea samples were observed for multiple comparison of means values of Br content (ppm.) between China, Korea, and Japanese *Undaria pinnatifidas *by the Ryan-Joiner testing method [12]. On the other hand, no differences were observed in I content between China and Korea and between Japan and Korea samples by the Ryan method (*P* < .03).

Bromine could not be completely dissolved and, further, Br has a tendency to vaporize in the acid decomposition sample preparation process for the ICP-MS. However in the case of X-ray fluorescence method, which is characterized by the rapid and easy handling, the above problem can be avoided and, hence, this could be a better and more promising method than ICP-MS. We earlier reported the rapidity and convenience of a discrimination method by near-infrared spectroscopy [3]. By combining the two different methods of NIR and X-ray fluorescence methods, which essentially give different organic and inorganic information, respectively, before the final method, ICP-MS method, a more convenient and rapid discrimination method can be developed. One of the main reasons for this is that NIR and X-ray fluorescence methods do not require that powder samples be prepared as solutions. We conclude that the above combination of methods could be used as a convenient discrimination method to meet the regulatory requirements as those of 2006 for the display of the production centers on *U. pinnatifida *in Japan. 

## 4. Conclusion

1. When using CART for the discrimination analysis on the production area from the elemental analysis results from the point of error rates, we take better discrimination results comparing to the LDA.
2. As one of convenient discrimination methods, we found the feasibility of using the elemental analysis, especially, the quantity of Br with the X-ray fluorescence analysis.

## Figures and Tables

**Table 1:** Elemental analysis results by ICP-MS (95 samples) (SNo. and ANo., respectively, mean sample No. and, Area No.).

No.	Area No.	Al	Ba	Ca	Fe	K	Mg	Mn	Sr	Li	Co	Ni	Cu	Zn	Rb	Y	Mo	Cd	La	Nd	Sm	Gd	W
1	1	5.4	1.94	1375	13.6	699	873	0.88	114	0.0542	0.01415	0.16683	0.98139	3.11705	0.17107	0.00649	0.02346	0.22912	0.00268	0.00247	0.00044	0.00079	0.00096
2	1	6.3	2.43	1613	12.8	891	944	0.78	153	0.05461	0.01331	0.17785	0.79213	4.7042	0.21672	0.01531	0.02205	0.2029	0.01335	0.00564	0.00135	0.0019	0
3	1	7.2	1.65	1452	11.7	1230	852	0.97	113	0.06378	0.01049	0.75033	0.43795	6.8553	0.29784	0.00631	0.01018	0.17076	0.00304	0.00294	0	0.00076	0
4	1	5.6	1.34	1223	9.5	1326	750	0.59	101	0.0509	0.00775	0.06355	0.65015	3.13791	0.27596	0.00712	0.0084	0.34223	0.003	0.0027	0	0.00069	0
5	1	5.7	1.35	1340	14.5	963	1003	1	90	0.09392	0.01273	0.09259	1.57583	3.29507	0.21745	0.0061	0.03727	0.22232	0.00232	0.00246	0.00069	0	0
6	1	5.4	1.56	1414	12.3	1461	829	0.89	123	0.0617	0.0146	0.12431	1.58335	5.42348	0.3309	0.01342	0.0922	0.2458	0.00394	0.0046	0.00149	0	0.00125
7	1	7	1.54	1353	13.3	1035	990	1.18	111	0.05292	0.0081	0.06942	0.68425	4.06385	0.24403	0.00611	0.01607	0.38852	0.00289	0.00246	0.0007	0.00066	0
8	1	9	1.85	1318	12.7	1573	752	0.62	115	0.06866	0.01585	0.17217	0.43662	6.68749	0.40428	0.0107	0.01152	0.09452	0.00438	0.00422	0.00101	0.00064	0
9	1	8.2	2.03	1311	18.7	992	803	0.75	114	0.08727	0.02529	0.17253	2.1749	4.32102	0.25587	0.01055	0.03569	0.0983	0.00573	0.0055	0.00129	0.00178	0.00071
10	1	7.5	2.03	1471	15.9	1152	929	1.02	132	0.06545	0.01874	0.14116	1.85378	6.64503	0.29321	0.00797	0.03239	0.33194	0.00291	0.00275	0.00074	0	0.00067
11	1	4.7	1.23	1277	9.2	726	901	0.37	82	0.08767	0.01343	0.07997	1.6429	7.32638	0.17385	0.00982	0.0366	0.03982	0.00311	0.00361	0.00126	0	0
12	1	5.6	1.78	1235	10.6	737	781	0.28	100	0.08199	0.01379	0.13274	1.38632	23.75906	0.18361	0.00784	0.06115	0.05717	0.00364	0.00339	0.00108	0	0
13	1	5	1.37	1435	12.7	1039	952	0.98	100	0.05738	0.01187	0.1929	0.71449	5.006	0.21873	0.00708	0.08483	0.20773	0.00229	0.00212	0.00047	0.00078	0
14	1	0	1.8	1210	8	1230	753	0	163	0.06234	0.01151	0.18377	0.8101	2.67774	0.26563	0.00998	0.00726	0.19707	0.0029	0.00319	0.00073	0.00135	0.00217
15	1	5	1.5	816	9	1100	613	0	101	0.05702	0.00836	0.12849	0.79145	2.43518	0.22074	0.00632	0.01818	0.28232	0.00198	0.00198	0.00055	0.00082	0
16	1	0	1.9	985	9	817	629	0	122	0.05764	0.01062	0.13431	0.76167	2.95464	0.16994	0.00923	0.02588	0.26033	0.00278	0.00354	0.00087	0.00098	0
17	1	0	1.6	896	4	814	694	1.04	120	0.05551	0.01118	0.12028	0.81748	2.91543	0.15053	0.00804	0.00416	0.20425	0.00257	0.00244	0.00095	0.00094	0
18	1	0	2	781	8	1300	597	0	127	0.06845	0.01301	0.13042	1.43438	2.96852	0.25851	0.01069	0.00633	0.19111	0.00308	0.00345	0.00097	0.00139	0
19	1	0	2.1	839	7	908	561	0.49	125	0.05997	0.02212	0.12225	0.87065	2.86427	0.18632	0.00846	0.01875	0.24961	0.00372	0.00362	0.00135	0.0012	0
20	1	0	2.1	882	6	1130	606	0.08	149	0.05353	0.01132	0.11277	0.80264	2.46142	0.22363	0.00922	0.01701	0.19042	0.00251	0.00308	0.00066	0.0012	0
21	1	0	2.1	818	8	1180	536	0.32	113	0.05824	0.01471	0.15687	1.07814	3.7452	0.24263	0.01357	0.01021	0.05977	0.00488	0.00531	0.00166	0.00197	0
22	1	9	1.8	818	9	802	546	0	95	0.0605	0.01308	0.11455	0.93649	4.27749	0.17262	0.00584	0.02282	0.03893	0.00304	0.0022	0.00043	0.00066	0
23	2	12.2	2.98	1111	13.9	1258	635	1.73	113	0.04433	0.01146	0.17701	0.83431	4.26196	0.32135	0.0251	0.01169	0.12784	0.00974	0.0099	0.00226	0.00256	0
24	2	18.6	7.86	1525	20.1	1291	716	1.88	126	0.05644	0.00967	0.19439	0.2221	5.59659	0.32056	0.02647	0.02549	0.2867	0.01819	0.01082	0.0024	0.00333	0
25	2	8.7	2.59	1398	11.4	1396	784	0.83	131	0.08849	0.018	0.22589	0.88814	6.89341	0.38449	0.02964	0.01278	0.05926	0.00947	0.00823	0.00332	0.00447	0.00681
26	2	15.2	2.17	1279	17.8	1449	661	0.93	106	0.06572	0.03901	0.16264	0.98079	8.28882	0.4012	0.01866	0.02158	0.07615	0.00936	0.00729	0.00206	0.00273	0.00336
27	2	6.8	1.37	1372	7.9	636	922	0.53	103	0.07893	0.0078	0.08874	1.61868	3.64924	0.18526	0.01715	0.02519	0.04093	0.00201	0.00002	0.00005	0.00095	0
28	2	8.5	2.42	1730	7.8	812	938	0.41	142	0.10557	0.01599	0.21756	1.91199	5.11889	0.29688	0.02938	0.04598	0.0459	0.00524	0.00228	0.00121	0.00186	0.0034
29	2	11.9	2.73	1402	14.9	1353	748	2.09	138	0.06916	0.02039	0.23734	1.79435	6.43411	0.39769	0.02846	0.02741	0.04046	0.01147	0.00775	0.00165	0.00252	0
30	2	7.9	2.43	1069	9.6	1866	623	0.85	114	0.0623	0.00853	0.14704	1.64154	6.43611	0.50854	0.02759	0.02128	0.05556	0.00604	0.00442	0.00116	0.00217	0
31	2	8.3	1.55	1029	7.2	871	623	0.53	87	0.05386	0.008	0.20293	2.05586	4.417	0.24011	0.02015	0.02597	0.04883	0.00387	0.00295	0.00053	0.00134	0
32	2	4.9	5.61	1067	10.7	1266	534	1.67	124	0.05655	0.0073	0.14379	0.81434	4.05703	0.29288	0.0212	0.01666	0.02969	0.00329	0.00229	0.00034	0.00173	0
33	2	11.9	2.84	1350	14.5	1338	790	1.1	134	0.06721	0.01104	0.17086	0.80628	8.03601	0.34309	0.02309	0.01488	0.03194	0.00799	0.00576	0.0014	0.0024	0
34	2	6.1	2.46	1280	10.3	1161	779	0.89	116	0.05909	0.008	0.14145	0.69256	6.03411	0.28334	0.0169	0.03505	0.03875	0.00412	0.003	0.00044	0.00153	0
35	2	11.3	2.26	1273	12	1038	795	1.1	114	0.06611	0.01134	0.19113	0.74343	4.93927	0.25781	0.0241	0.01832	0.05149	0.00773	0.00624	0.00165	0.00249	0
36	2	3	1.9	884	9	880	658	0.23	126	0.06874	0.00572	0.17648	1.21315	3.83684	0.1662	0.01877	0.01821	0.03363	0.00337	0.00369	0.00088	0.00122	0.0025
37	2	3	2	821	9	1090	623	0.39	119	0.05392	0.00349	0.12209	1.03493	2.96145	0.19666	0.01185	0.02581	0.02706	0.00296	0.00288	0.00036	0.00096	0.00215
38	2	2	6.7	590	11	1010	368	1.77	114	0.04494	0.00614	0.16751	1.49357	3.79587	0.16605	0.01089	0.00693	0.02164	0.0027	0.00293	0.00058	0.0011	0.00278
39	2	0	5.7	643	12	903	399	2.24	110	0.04365	0.00344	0.17123	1.54981	2.81897	0.15579	0.01053	0.01715	0.02292	0.00261	0.00242	0.00047	0.00057	0.00301
40	2	3	1.7	822	9	888	572	0.28	98	0.04799	0.00523	0.12915	1.03287	3.38812	0.16227	0.0126	0.00616	0.02484	0.00368	0.0032	0.00069	0.00133	0
41	2	0	1.7	728	10	892	625	0.46	97	0.05638	0.01156	0.14221	1.01484	3.64588	0.17658	0.01608	0.01765	0.03393	0.00616	0.00597	0.00137	0.00173	0.00174
42	2	2	1.7	648	8	1210	544	1.12	105	0.05117	0.00679	0.11756	0.77304	4.29234	0.22612	0.01294	0.01473	0.03425	0.00333	0.00292	0.00088	0.00131	0.00108
43	2	0	2	760	9	1050	564	0.36	120	0.05483	0.01156	0.16162	0.95856	4.129	0.21756	0.01294	0.03057	0.03252	0.00369	0.00332	0.00096	0.0019	0.00151
44	2	3	1.9	779	8	1250	587	0.53	126	0.05176	0.00751	0.13718	0.95634	3.11655	0.25131	0.01359	0.02696	0.03482	0.0042	0.0039	0.00106	0.00164	0
45	2	0	2.3	822	9	1400	627	0.69	128	0.06677	0.01311	0.18294	0.94396	4.19777	0.29856	0.01513	0.01533	0.03218	0.00471	0.00402	0.00116	0.00213	0.00272
46	2	0	1.9	781	7	1190	554	0.48	104	0.0619	0.00771	0.14762	0.85024	3.22837	0.23842	0.01913	0.01217	0.03268	0.00478	0.00538	0.00131	0.00175	0
47	2	2	2.4	912	12	1440	518	0.49	116	0.05604	0.02853	0.22684	0.86944	4.98316	0.31686	0.01701	0.01694	0.03375	0.0086	0.0072	0.00178	0.00234	0
48	3	32.7	7.15	1334	30.1	1862	965	2.91	120	0.08515	0.01802	0.16843	0.98543	7.20925	0.46605	0.03165	0.02004	0.20342	0.02111	0.02136	0.00393	0.00598	0.00143
49	3	34.8	6.7	1005	29.3	1787	776	1.6	105	0.08669	0.0407	0.28747	1.37302	7.39241	0.45882	0.04273	0.01546	0.29256	0.0378	0.03096	0.00634	0.00787	0
50	3	117.5	7.25	1163	41.6	1464	861	1.61	101	0.11691	0.02487	0.16076	1.20548	5.82687	0.55349	0.05435	0.02448	0.59295	0.05972	0.0537	0.01124	0.01217	0.00379
51	3	25.5	6.85	1213	25.9	1636	945	1.72	116	0.08874	0.01833	0.14832	0.97414	4.17426	0.44464	0.02311	0.01486	0.24471	0.0271	0.02152	0.00361	0.00554	0
52	3	57	8.72	1564	55.1	1251	995	2.51	137	0.08881	0.02508	0.19936	1.08124	6.73134	0.35813	0.04524	0.0449	0.17647	0.05455	0.04117	0.00774	0.00883	0.00115
53	3	150	8.23	1214	88.1	2182	918	3.39	113	0.17248	0.03916	0.22137	1.12308	6.99515	0.73917	0.06461	0.02338	0.24762	0.10389	0.08367	0.0161	0.01629	0.00597
54	3	32.1	5.95	1211	25.3	1686	778	1.89	114	0.06799	0.02094	0.21172	1.17071	8.00261	0.45272	0.04327	0.01167	0.45391	0.0186	0.01753	0.00392	0.00645	0
55	3	54.8	6.24	1617	37.1	1542	1095	3.32	127	0.08071	0.0153	0.08068	0.25295	7.38076	0.40822	0.03819	0.01768	0.43595	0.0274	0.02612	0.00594	0.00548	0
56	3	35.5	10.15	1625	40.3	1783	919	2.93	159	0.06886	0.02878	0.29481	0.45887	6.21704	0.46524	0.04491	0.02449	0.23176	0.03027	0.02756	0.00578	0.00658	0
57	3	66.4	6.97	1710	44.8	1242	1210	2.76	115	0.0911	0.01898	0.13521	1.25059	7.7665	0.38781	0.04559	0.01794	0.37026	0.04611	0.04124	0.00811	0.008	0
58	3	20.7	6.07	1459	22.6	1541	1005	2.98	113	0.06192	0.01324	0.15806	0.81674	5.92709	0.40255	0.02623	0.01184	0.31727	0.01897	0.01859	0.00432	0.00376	0
59	3	40.5	6.37	1080	29	1906	823	0.87	118	0.07643	0.02911	0.31762	0.74707	6.59626	0.56804	0.03725	0.01136	0.33082	0.02929	0.0301	0.00552	0.00602	0
60	3	181.9	7.15	1577	80.9	1603	1467	2.94	117	0.08301	0.01483	0.12122	0.73307	5.6655	0.43486	0.03059	0.01647	0.3218	0.02953	0.02576	0.0054	0.00507	0
61	3	147.3	7.29	1413	93.5	1785	855	3.4	116	0.16342	0.03993	0.27427	1.00277	4.71714	0.68076	0.07062	0.01449	0.38032	0.09272	0.08081	0.01582	0.01409	0
62	3	142.8	7.78	1747	83.2	1792	871	3.23	131	0.15818	0.04547	0.26347	0.89004	4.05333	0.68924	0.07297	0.01723	0.33105	0.09484	0.08197	0.01682	0.01498	0
63	3	21.9	6.14	1179	21.3	1688	786	2.26	109	0.05533	0.0157	0.15839	0.92013	6.77109	0.43631	0.02756	0.01873	0.41478	0.01648	0.01511	0.00366	0.00349	0
64	3	54.3	7.04	1389	36.6	1651	925	2.75	114	0.0478	0.01503	0.13907	0.84562	5.23622	0.44087	0.03699	0.0264	0.37085	0.04415	0.0376	0.0072	0.00704	0
65	3	42.8	6.39	1629	34.3	1681	811	1.98	136	0.06881	0.01577	0.14314	0.72471	4.41137	0.44554	0.02753	0.02663	0.22586	0.03893	0.03515	0.00585	0.00565	0
66	3	83.2	7.8	1578	38.5	1733	1052	3	121	0.08926	0.01389	0.08352	0.66891	4.23632	0.52241	0.04216	0.00819	0.29782	0.04822	0.0444	0.00869	0.00741	0
67	3	32	7.5	921	34	1690	567	1.85	116	0.06804	0.05074	0.19172	0.75233	4.27229	0.37451	0.02883	0.04509	0.16358	0.02428	0.02396	0.00553	0.00516	0.01321
68	3	59	5	725	23	1300	489	1.53	81	0.07487	0.01577	0.169	0.64346	3.49819	0.34244	0.03497	0.02044	0.34333	0.04851	0.04418	0.00855	0.00735	0.00081
69	3	35	6.2	938	21	1570	618	1.5	106	0.06654	0.02335	0.20623	0.58988	5.0014	0.35409	0.02861	0.01413	0.15892	0.03247	0.02475	0.00464	0.0053	0
70	3	33	5.8	879	23	1300	619	1.31	85	0.06727	0.0133	0.15309	0.73361	4.52006	0.30275	0.02317	0.01428	0.30424	0.02288	0.02018	0.00495	0.004	0
71	3	22	4.7	639	16	1550	552	1.29	77	0.08278	0.02069	0.2328	0.83111	4.76853	0.37043	0.03226	0.01232	0.40566	0.02722	0.02373	0.00526	0.00474	0
72	3	17.3	5.99	840	16.2	1130	780	1.48	83	0.08994	0.01191	0.17059	0.95605	2.80027	0.26287	0.01951	0.02006	0.33315	0.01446	0.01284	0.00271	0.00287	0
73	3	67.4	6.97	787	40.7	1140	758	2.51	84	0.10418	0.03571	0.32161	1.05128	3.72228	0.33374	0.0406	0.01956	0.16176	0.04212	0.04008	0.00878	0.00812	0
74	3	57.7	6.57	1110	31.7	1400	783	1.97	108	0.09485	0.01686	0.16481	0.772	3.16761	0.41493	0.03786	0.0116	0.32401	0.06511	0.06057	0.01058	0.0094	0.00151
75	3	24.8	5.99	1220	24.4	1800	739	1.43	123	0.08695	0.0161	0.41791	0.87835	4.67514	0.4484	0.03013	0.01885	0.31946	0.0519	0.04366	0.00866	0.00719	0
76	3	41.5	5.68	928	31.5	1580	665	1.04	99	0.09737	0.02702	0.26321	0.88837	5.33564	0.42419	0.03304	0.01086	0.36017	0.03244	0.03105	0.00667	0.0063	0
77	4	6	2.1	750	9.9	879	450	0.93	76	0.03965	0.00969	0.09892	2.32801	5.9116	0.20272	0.01218	0.01676	0.12467	0.00478	0.00455	0.00092	0.00125	0
78	4	67.5	6.2	1315	38.1	1665	961	1.75	108	0.11877	0.02577	0.24007	1.72561	5.98751	0.55625	0.04559	0.01487	0.38436	0.05218	0.04104	0.00858	0.00749	0.00193
79	4	15.1	2.5	1179	19.9	2001	713	1.46	124	0.07429	0.01553	0.36134	1.43287	4.45066	0.57157	0.01768	0.02508	0.11813	0.01407	0.01183	0.00316	0.00327	0.00099
80	4	14.6	2.38	1166	37.6	1724	719	1.75	126	0.0722	0.01691	0.02697	2.36	4.95932	0.50072	0.01647	0.02117	0.139	0.00863	0.0072	0.00149	0.00203	0
81	4	15.1	2.87	1282	18.9	1845	735	1.17	138	0.07857	0.0264	0.1218	1.82778	5.22642	0.55782	0.02206	0.02545	0.16504	0.01266	0.01066	0.00219	0.00307	0
82	4	23.4	2.21	1098	20.6	1769	723	1.24	119	0.0809	0.01554	0.03717	1.84797	4.40811	0.55019	0.01926	0.02201	0.1579	0.01658	0.0136	0.00285	0.00331	0
83	4	7.5	2.92	1477	21.5	1184	1014	1.6	152	0.06253	0.01484	0.07442	2.29462	5.77201	0.31975	0.01384	0.03589	0.12925	0.0093	0.00688	0.00174	0.00173	0
84	4	7.5	2.69	1420	21.3	1114	983	1.43	141	0.05248	0.0112	0.0773	0.78883	5.12785	0.30649	0.01227	0.02812	0.13589	0.00882	0.00889	0.0019	0.00179	0
85	4	18.4	2.1	1171	21.6	1771	759	1	109	0.06616	0.0132	0.11224	0.78616	4.77861	0.48312	0.01443	0.01797	0.20485	0.01257	0.01067	0.00226	0.00189	0
86	4	9.3	2.54	1472	21.5	1601	898	1.51	140	0.0594	0.01326	0.22647	0.94003	5.24577	0.43846	0.01641	0.02387	0.08145	0.00937	0.00799	0.00201	0.00236	0
87	4	8.9	2.76	1457	18.6	1583	886	1.5	145	0.05628	0.01191	0	0.81296	5.44063	0.42609	0.01339	0.01762	0.08327	0.00685	0.0056	0.00602	0.00141	0
88	4	11.4	2.57	1452	19.2	1666	863	1.48	139	0.05882	0.01628	0.03643	0.84429	5.78241	0.44012	0.01713	0.02079	0.0729	0.00811	0.00731	0.00184	0.00181	0
89	4	23.4	2.63	1363	26	2009	758	1.49	147	0.07748	0.01941	0.24689	0.88104	5.12101	0.57246	0.023	0.02062	0.10381	0.01911	0.0165	0.00402	0.00431	0
90	4	10.3	2.51	1306	17.5	1811	766	1.84	138	0.05733	0.01299	0.14919	0.67941	4.80655	0.47569	0.01586	0.03141	0.17062	0.01073	0.00989	0.00227	0.00226	0
91	4	27.8	3.15	1217	31.2	1726	736	1.73	126	0.07836	0.02027	0.11442	0.62579	4.99679	0.47799	0.02137	0.02146	0.27568	0.02566	0.02015	0.00432	0.00379	0
92	4	48	4.17	1190	37	1319	742	1.32	124	0.09968	0.02134	0.19624	1.18233	3.00584	0.36042	0.02267	0.02508	0.14795	0.02287	0.02187	0.00442	0.00221	0.00094
93	4	11	3.6	1439	22	1451	849	1.81	145	0.05659	0.01007	0.12401	0.76767	3.5202	0.33506	0.01434	0.02182	0.12254	0.00766	0.0072	0.0015	0.00176	0
94	4	5	2	921	9	1320	621	0.78	144	0.05918	0.01743	0.16071	1.5454	3.13996	0.3301	0.01822	0.01719	0.11988	0.0101	0.01016	0.00274	0.00281	0
95	4	15	4.3	611	15	1330	428	0.69	73	0.05668	0.01731	0.16661	1.19903	4.80615	0.31385	0.0224	0.01336	0.35779	0.01253	0.01257	0.00294	0.00321	0

Area No.1 sanriku (Japan), 2 naruto (Japan), 3 China, 4 Korea.

**Table:** (a) By nation (*n* = 3)

Production correct		Japan	China	Korea
Center	Ratio (%)			
Japan	93	44	1	2
China	100	0	29	0
Korea	74	3	2	14

**Table:** (b) By area (*n* = 4)

Production correct		Sanriku	Naruto	China	Korea
Center	Ratio (%)				
Sanriku	87	19	2	0	1
Naruto	92	0	23	1	1
China	100	0	0	29	0
Korea	74	2	1	2	14

**Table:** (a) By nation (*n* = 3)

Production correct		Japan	China	Korea
Center				
Ratio (%)
Japan	100	47	0	0
China	100	0	29	0
Korea	74	1	1	17

**Table:** (b) By area (*n* = 4)

Production correct		Sanriku	Naruto	China	Korea
Center	Ratio (%)				
Sanriku	100	22	0	0	0
Naruto	92	0	23	1	1
China	100	0	0	29	0
Korea	74	2	0	1	16
